# Synthesis, characterization and observation of antisite defects in LiNiPO_4_ nanomaterials

**DOI:** 10.1038/srep11041

**Published:** 2015-06-19

**Authors:** Murukanahally Kempaiah Devaraju, Quang Duc Truong, Hiroshi Hyodo, Yoshikazu Sasaki, Itaru Honma

**Affiliations:** 1 Institute of Multidisciplinary Research for Advanced Materials, Tohoku University, Katahira 2-1-1, Aoba-ku, Sendai 980-8577, Japan; 2 DATUM Solution Business Operations, JEOL, Ltd., Tokyo 196-0022, Japan

## Abstract

Structural studies of high voltage cathode materials are necessary to understand their chemistry to improve the electrochemical performance for applications in lithium ion batteries. LiNiPO_4_ nanorods and nanoplates are synthesized via a one pot synthesis using supercritical fluid process at 450 ^o^C for 10 min. The X-ray diffraction (XRD) analysis confirmed that LiNiPO_4_ phase is well crystallized, phase purity supported by energy dispersive spectroscopy (EDS) and elemental mapping by scanning electron transmission electron microscopy (STEM). For the first time, we have carried out direct visualization of atom-by-atom structural observation of LiNiPO_4_ nanomaterials using high-angle annular dark-field (HAADF) and annular bright-field (ABF) scanning transmission electron microscopy (STEM) analysis. The Rietveld refinement analysis was performed to find out the percentage of antisite defects presents in LiNiPO_4_ nanoplates and about 11% of antisite defects were found. Here, we provide the direct evidence for the presence of Ni atoms in Li sites and Li in Ni sites as an antisite defects are provided for understanding of electrochemical behavior of high voltage Li ion battery cathode materials.

Development of high energy storage systems are necessary to fulfill the energy demand of the world and also to solve the problems related to climate change and shortage of fossil fuels. At present, the research on investigation of next generation of electrode materials are a hot research topic after the successful commercial application of LiCoO_2_. Lithium-ion batteries are considered as cheap energy storage technology because they offer great energy storage systems and they show promising applications in hybrid electric vehicles, portable electronics and electric vehicles^1^,^2^,^3^,^4^,^5^. There is a need of environmentally friendly, safe and cheap cathode materials for application in lithium ion battery. Cathode materials are important component of lithium ion batteries; there are varieties of cathode materials available till now, among them olivine structured materials are also considered as cheap and promising cathode materials for application in Li-ion batteries^1^,^2^,^3^,^4^,^5^. Among the olivine structured cathode materials (LiMPO_4_ (M=Fe, Mn, Co and Ni), LiCoPO_4_ and LiNiPO_4_ are considered as high voltage cathode materials. However, the practical use of LiCoPO_4_ and LiNiPO_4_ are at the moment barred due to its poor cyclic performances because of intrinsic low electronic conductivity, limited lithium diffusion and another possible reason is due to the electrolyte degradation at higher voltage. There has been moderate development was achieved concern to LiCoPO_4_ cathode materials when compared to LiNiPO_4_ cathodes. Due to its discharge voltage plateau around 5.1 V and its large capacity of 170 mAhg^−1^, LiNiPO_4_ is an interesting high voltage cathode materials, at the moment its electrochemical performances is not properly investigated using presently available electrolytes^6^.

Recently, synthesis and characterization of LiNiPO_4_ materials using different synthesis route have been reported and few of them reported moderate electrochemical performances^7^,^8^,^9^,^10^,^11^,^12^. It has been reported that, controlled size and morphology of cathode materials could improve the electrochemical performances^1^,^13^,^14^. To control the shape and morphology of cathode materials solution based synthesis is more suitable^1^. Recently, we have reported size and morphology controlled synthesis of variety of cathode materials such as phosphate, silicates and flurophosphates via a supercritical fluid process^15^,^16^,^17^,^18^,^19^,^20^,^21^,^22^,^23^ and observe the improvement of electrochemical performances with related to size and shape.

Herein, we report synthesis and characterization of LiNiPO_4_ nanoplates prepared via supercritical fluid process. Attempt has been made to investigate the presence of antisite defects in LiNiPO_4_ cathode materials, which is also a kind of reason for low capacity issues in high voltage cathodes^24^,^25^,^26^.

## Results and Discussion

### Synthesis and powder X-ray diffraction analysis

**Figure 1:**
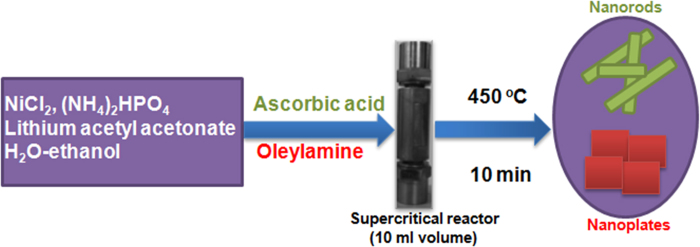
Schematic illustration of LiNiPO_4_ nanorods-nanoplates synthesis via supercritical fluid process.

Using supercritical fluid process we achieved direct synthesis of phase pure LiNiPO_4_ due to the overwhelming advantages of this process as we reported in many of our previously published papers^15^,^16^,^17^,^18^,^19^,^20^. The synthesis procedure for LiNiPO_4_ cathode materials is shown in Fig. 1. Using same starting materials and by changing reducing agents, LiNiPO_4_ with two kinds of morphologies were synthesized.

**Figure 2:**
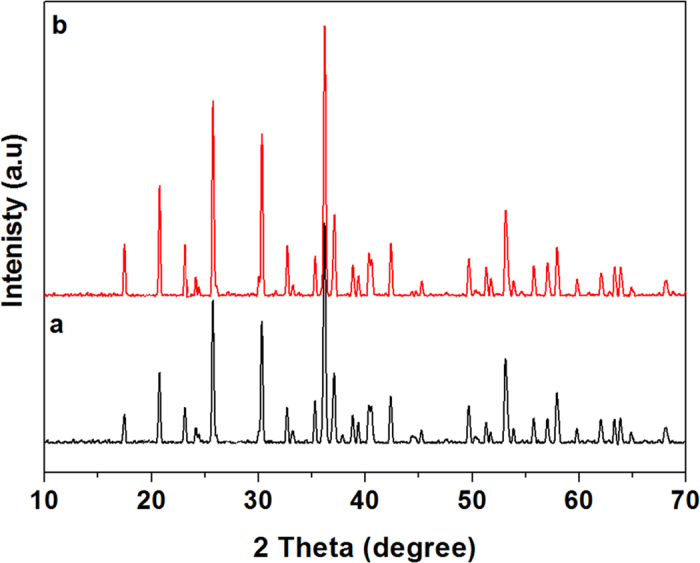
XRD pattern of as-synthesized LiNiPO_4_ at 450 °C for 10 min using (**a**) ascorbic acid and (**b**) oleylamine as reducing agent/surfactant via supercritical fluid process.

The XRD pattern of as-synthesized LiNiPO_4_ cathode material at 450 ^o^C for 10 min using ascorbic acid and oleylamine are shown in Fig. 2. The crystal structures of as-synthesized materials are identified as LiNiPO_4_ and all of reflections are indexed to orthorhombic crystal system and belongs to *Pnma* space group. From the XRD pattern, it is evident that single phase of LiNiPO_4_ was successfully synthesized without any impurities. The two samples showed similar XRD pattern but variations in their peak intensities, where LiNiPO_4_ synthesized using oleylamine (Fig. 2b) showed slightly higher intensity than the LiNiPO_4_ synthesized using ascorbic acid (Fig. 2a).

### Morphologies and size of LiNiPO4 particles

**Figure 3:**
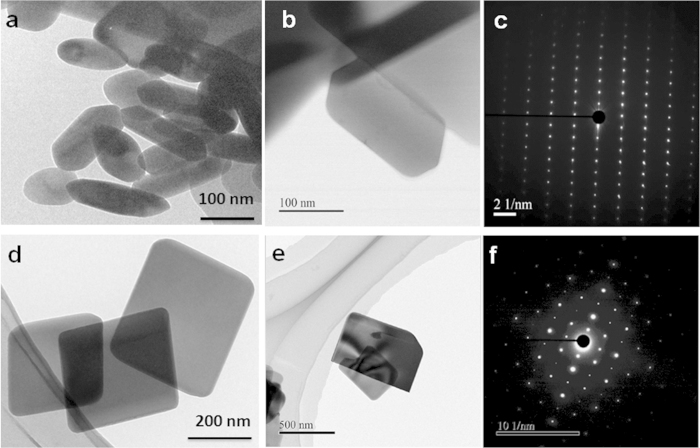
TEM HRTEM and SAED images of as-synthesized LiNiPO_4_ at 450 °C for 10 min using (**a**) ascorbic acid (Fig. 3(a–c)) and (**b**) oleylamine (Fig. 3(d–f)) as reducing agent/surfactant via supercritical fluid process.

The as-synthesized LiNiPO_4_ particles were analyzed using TEM and HRTEM analysis as shown in Fig. 3. LiNiPO_4_ particles synthesized using ascorbic acid and oleylamine as reducing agents showed rod and plate like morphologies. The rod like LiNiPO_4_ exhibit particle size from 100–200 nm in length, 50–80 nm in diameter as shown in Fig. 3a,b. The plate like LiNiPO_4_ exhibit particles size from 250–400 nm in length, 300–600 nm in width, and side thickness of less than 20 nm as shown in Fig. 3d,e. The selected area diffraction pattern taken along [010] axis of rod and plate like particles shown in Fig. 3c,f, confirms that the synthesized LiNiPO_4_ are single crystalline in nature. The diffraction pattern is consistent with morphologies of LiNiPO_4_ nanorods and nanoplates.

The rod like LiNiPO_4_ was obtained in the presence of water-ethanol mixed solvents, where enormous amount of hydroxyl ions are released during crystallization of LiNiPO_4_, which promote one dimensional growth of LiNiPO_4_ particles. The ascorbic acid is just worked as reducing agents and not as surfactant. But oleylamine act both as reducing agent and surfactant, that’s why plate like particles are obtained using oleylamine, where oleylamine capped on to the specific crystal planes and allows LiNiPO_4_ to grow on other specific planes. In the case of plate like morphology, oleylamine capping on *b*-axis, so that, we obtain plate like particles with less than 20 nm in diameter along *b*-axis. We have also observed this phenomenon in the synthesis of LiCoPO_4_ nanoplates under supercritical conditions^21^.

### EDS and elemental mapping of LiNiPO4 nanorods and nanoplates

**Figure 4:**
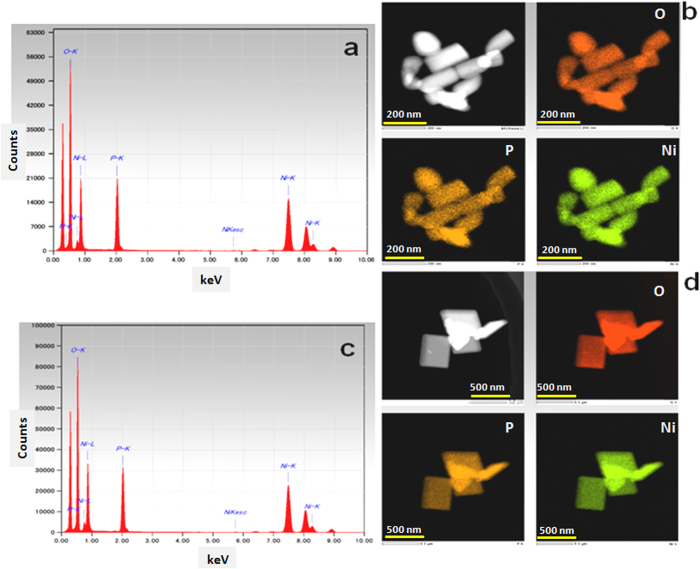
EDS and elemental mapping images of as-synthesized LiNiPO_4_ at 450 °C for 10 min using (**a**) ascorbic acid (Fig. 4(a,b)) and (**b**) oleylamine (Fig. 4(c,d)) as reducing agent/surfactant via supercritical fluid process.

The purity of LiNiPO_4_ nanorods and nanoplates were confirmed by STEM analysis, Fig. 4a,b shows the EDS spectra of LiNiPO_4_ nanorods and nanoplates, in both the spectra the presence of all the elements such as O, P, and Ni were present and no other impurity was observed, both XRD and EDS supports the purity of LiNiPO_4_ nanorods and nanoplates. Furthermore, the elemental mapping was carried out for LiNiPO_4_ nanorods and nanoplates, the homogeneous distribution of oxygen, phosphor and nickel elements were clearly observed as shown in Fig. 4c,d.

### Antisite defects in LiNiPO4 nanomaterials

The structural observation using HAADF/ABF-STEM analysis show the presence of antisite defects in olivine structured cathode materials. So far, antisite defects in LiFePO_4_, LiMnPO_4_ and LiCoPO_4_ have been reported^24^,^25^,^26^,^27^,^28^.

**Figure 5:**
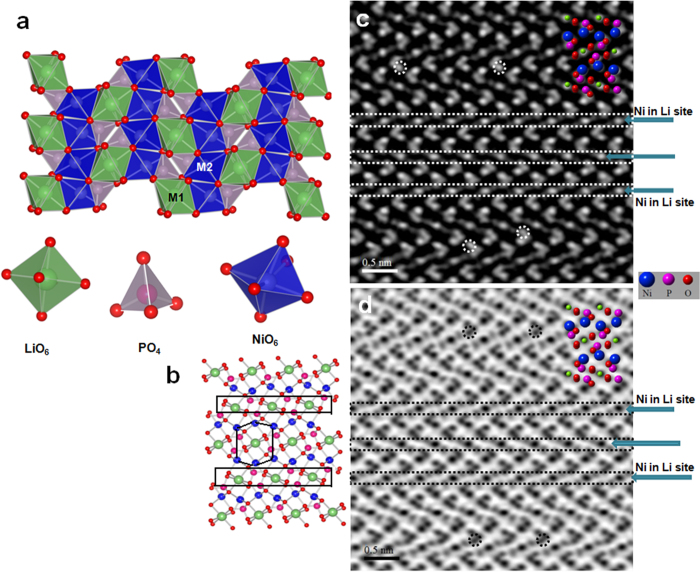
(**a**) and (**b**) Crystal structure of LiNiPO_4_ illustrating well ordered cation partitioning of Li, Ni, P and O atoms along [010] direction, showing Ni at M2 site and Li at M1 site in the structure **b**) Crystal structure of LiNiPO_4_ illustrating that six Ni atoms make hexagon and showing the lithium columns (square mark). **c** and **d**) HAADF and ABF-STEM images viewed along [010] direction, showing the presence of Ni in Li site as antisite defects (See dotted squares and arrow marks) and low contrast at Ni site due to presence of Li in Ni site (dotted circle mark).

However, there is no report available on reporting antisite defects in LiNiPO_4_ cathodes, and for the first time we have observed antisite defects in LiNiPO_4_ nanomaterials. LiNiPO_4_ crystal structure is composed of slightly distorted NiO_6_ octahedra, P ions are located at the center of PO_4_ tetrahedra. Both the lithium and nickel ions occupy the octahedral sites, lithium is located at edge-sharing M1 sites and Ni is located at corner sharing M2 sites in LiNiPO_4_ structure as shown in Fig. 5a. It has been reported that, cation exchange will occur between the two octahedral M sites in olivine structured cathode materials as antisite defects^26^,^27^,^28^,^29^,^30^,^31^,^32^.

Figure 5c,d shows the HAADF-STEM and ABF-STEM image viewed along [010] crystal direction of olivine structured plate like LiNiPO_4_ cathode nanomaterials synthesized supercritical fluid process at 450 ^o^C for 10 min of reaction time.

For comparison, two dimensional atomic arrangement of a unit cell structure is superimposed on HAADF-STEM and ABF-STEM image. The bright and dark contrast produced by Ni atoms can be clearly observed in HAADF image and ABF image as shown in Fig. 5b,c. Phosphor atoms are located neighboring to each Ni atoms, which produce low dark and bright contrasts compared to that of Ni atoms. In a unit cell, six Ni atoms form each other a hexagon configuration can be seen in Fig. 5b.

Due to the overlapping of three atomic columns when viewed along [010] projections O columns are not well resolved compared to Ni and P atoms, which is well agreement with the observation of LiFePO_4_ crystal structure and LiCoPO_4_ crystal structures^26^,^32^. In HAADF mode, Li atoms are invisible and no contrast could be found along Li columns in by HAADF mode^26^,^27^,^28^. However, the bright and dark contrast were observed along Li columns, which clearly indicates that Ni atoms are moved from M2 site to M1 sites and occupy the Li sites (see the dotted square arrow mark), which results in weak contrasts of some Ni columns as shown in Fig. 5c,d. When compared to LiFePO_4_, LiMnPO_4_ and LiCoPO_4_ structures^26^,^27^,^28^, Li to Ni exchange as an antisite defects in LiNiPO_4_ are higher as they exhibit very strong contrast and are homogeneously distributed. In addition, some of the Ni atoms are occupied by Li atoms, which could be noticed due to the weak bright and dark contrast observed at Ni sites (see the circle mark). During electrochemical reaction, the Li ions diffusion through [010] direction are blocked by Ni atoms which results in low discharge capacity of olivine structured cathode materials. The antisite defects are usually occurred in olivine cathode materials synthesized at low temperatures. The low electrochemical performance of high voltage olivine structured materials such as LiCoPO_4_ and LiNiPO_4_ are due to low electronic conductivity, lack of high voltage electrolytes and also due to the presence of antisite defects.

Further, Rietveld refinement analysis was carried out for LiNiPO_4_ nanoplates to support the STEM observation of antisite defects and to mention quantitatively the amount of antisite defects. Table 1 shows the parameters obtained from Rietveld refinement analysis. The refined cell parameters of LiNiPO_4_ nanoplates are *a* = 10.0330(4) Å, b = 5.8528(2) Å, and c = 4.6767(2) Å. The refinement analysis showed approximately 5% of Ni in Li site (4*a* site) and 5% of Li in Ni site (4*c* site), total 10% of antisite defects are found in LiNiPO_4_ nanoplates synthesized via supercritical fluid process. Chung *et al.*^26^ have shown around 1% of antisite defects by Rietveld analysis for LiFePO_4_ synthesized at 600 ^o^C and 15% of antisite defects by using quantitative STEM for the same sample. So that, there is difference between antisite defects observation experimentally and by Rietveld refinement. Our STEM observation of LiNiPO_4_ showed high percentage of antisite defects as we observed high contrast in lithium columns and low contrast at Ni site. It has been reported that, the antisite defects could increase upon electrochemical cycling, this phenomenon was observed for LiCoPO_4_ after few cycles and the pristine sample had 5% of antisite defects^33^. High percentage of antisite defects can be expected for olivine structured materials synthesized at low temperature solution process with nanometer scale.

LiNiPO_4_ cathode nanomaterials with nanorods and nanoplates like morphologies were successfully synthesized via one pot synthesis route using supercritical fluid process. The pure phase and phase purity of LiNiPO_4_ nanorods and nanoplates were confirmed by XRD and EDS analysis. The rod like LiNiPO_4_ exhibited particle size from 100–200 nm in length, 50–80 nm in diameter and the plate like LiNiPO_4_ exhibited particles size from 250–400 nm in length, 300–600 nm in width, and side thickness of less than 20 nm. Further, LiNiPO_4_ nanoplates were analyzed by HAADF-STEM and ABF-STEM analysis to observe the structure of LiNiPO_4_ crystals. The presence of Ni and P atoms are observed with bright and dark contrast. As expected, Ni atoms are found to occupy Li sites as the antisite defects at Li sites and Li in Ni sites. The strong contrast at Li sites confirms the movement of Ni atoms from M2 sites to M1 sites. The Rietveld refinement analysis showed approximately 10% of antisite defects. The antisite distributions are homogeneous and they are probably unavoidable in the olivine cathode materials synthesized at low temperatures.

## Methods

LiNiPO_4_ nanorods and nanoplates were synthesized from NiCl_2_, (Wako, Japan) (NH_4_)_2_HPO_4_ (Wako, Japan) and lithium acetyl acetonate (Wako, Japan) in 1:1:1 molar ratio. Oleylamine (Wako, Japan) was used both as surfactant and reducing agent and Ascorbic acid (Wako, Japan) used reducing agent. First, NiCl_2_.6H_2_O was dissolved in a solution of water-ethanol mixed solvents (1:1 volume ratio) and (NH_4_)_2_H_2_PO_4_ was added slowly with constant stirring followed by addition of lithium acetyl acetonate after that ascorbic acid or oleylamine (metal ion to surfactant 1:20) was added. The solution mixture was stirred for about few min after that 5 ml solution was transferred to batch reactors (4 reactors, each 10 ml volume). The batch reactors were heated at 450 ^o^C for 10 min and then reactors were quenched in cold water. The products were recovered by washing and dried in a vacuum for overnight.

### Material characterization

The powder X-ray diffraction (XRD) analysis was carried out using a Bruker AXS D8 Advance instrument with Cu Kα radiation. The XRD pattern was analyzed by the Rietveld method using the program RIETAN^34^ The morphology and size of the particles were determined using high-resolution transmission electron microscopy, High angle annular dark field (HAADF) images, elemental mapping and energy dispersive spectroscopy (EDS) were observed using JEM-2010F instrument equipped with a spherical aberration corrector (CEOS) at 200 KeV. The camera length was 6 cm; the BF aperture was 3cm, and HAADF and ABF detectors spanned the ranges of 70–180 and 12–24 mrad, respectively.

## Additional Information

**How to cite this article**: Devaraju, M. K. *et al.* Synthesis, characterization and observation of antisite defects in LiNiPO_4_ nanomaterials. *Sci. Rep.* **5**, 11041; doi: 10.1038/srep11041 (2015).

## Figures and Tables

**Table 1:** Parameters obtained from Rietveld refinement of the diffraction pattern of LiNiPO_4_ nanoplates, g is the occupancy of atoms.

Atom	Site	g	x	y	z
Li/Ni	4*a*	0.950(4)/0.050	0	0	0
Ni/Li	4*c*	0.950(7)/0.050	0.2759(2)	1/4	0.9856(6)
P	4*c*	1	0.0948(5)	1/4	0.427(2)
O1	4*c*	1	0.086(2)	1/4	0.731(3)
O2	4*c*	1	0.448(2)	1/4	0.247(3)
O3	8*d*	1	0.1589(9)	0.030(2)	0.257(2)

*a* = 10.0330(4) Å, *b* = 5.8528(2) Å, *c* = 4.6767(2) Å, *V* = 274.62(2) Å^3,^ *R*_wp_ = 28.3, *R*_B_ = 13.0, *R*_F_ = 8.6.
